# The role of interconnectivity in control of an Ebola epidemic

**DOI:** 10.1038/srep29262

**Published:** 2016-07-07

**Authors:** J. C. Blackwood, L. M. Childs

**Affiliations:** 1 Department of Mathematics and Statistics Williams College, Williamstown, MA 01267, USA; 2 Center for Communicable Disease Dynamics Harvard T.H. Chan School of Public Health, Boston, MA 02115, USA

## Abstract

Several West African countries - Liberia, Sierra Leone and Guinea - experienced significant morbidity and mortality during the largest Ebola epidemic to date, from late 2013 through 2015. The extent of the epidemic was fueled by outbreaks in large urban population centers as well as movement of the pathogen between populations. During the epidemic there was no known vaccine or drug, so effective disease control required coordinated efforts that include both standard medical and community practices such as hospitalization, quarantine and safe burials. Due to the high connectivity of the region, control of the epidemic not only depended on internal strategies but also was impacted by neighboring countries. In this paper, we use a deterministic framework to examine the role of movement between two populations in the overall success of practices designed to minimize the extent of Ebola epidemics. We find that it is possible for even small amounts of intermixing between populations to positively impact the control of an epidemic on a more global scale.

From late 2013, throughout 2014 and stretching into 2015, the West African countries of Sierra Leone, Guinea, and Liberia experienced the most devastating Ebola virus epidemic to date^1^,^2^. While previous outbreaks have been primarily confined to remote villages, this outbreak was much larger in part due to the arrival of the Ebola virus in large population centers^3^. Consequently, there were nearly 30,000 suspected cases and over 11,000 deaths over the course of the epidemic, the majority of which occurred in Liberia^2^. Control of the outbreak was further hindered by the spread of the virus in three neighboring countries with different public health agendas, varying levels of resources, and, at times, inconsistent strategies for treating of the disease^4^,^5^,^6^.

Successful control of the outbreak in western Africa relied on multifaceted approaches that implement both standard practices for treating cases (e.g. hospitalization) as well as community interventions (e.g. safe burial practices)^1^. Previous modeling studies have explored the dynamical impact of such management strategies on the Ebola epidemic in order to determine the most effective policies (reviewed in ref. 7). In addition to hospitalization and safe burial practices, mathematical models have investigated the effects of increasing the number of available beds in hospitals, expanding the number of Ebola treatment units (ETUs), accelerating case identification, and implementing a quarantine policy for infected individuals (e.g. ref. 8, 9, 10, 11, 12, 13, 14, 15). However, these studies have assumed that treatment and prevention methods are implemented homogeneously within a population or introduce them within stochastic models of disease spread (e.g. ref. 16, 17, 18, 19). Here, we determine the effectiveness of interventions when interacting populations implement policies that have not been coordinated.

Specifically, we consider a two-patch model that is connected by varying levels of movement between patches. Each patch alone may employ some combination of hospitalization, quarantine or enhanced burial safety to mitigate the spread of infections, but the policy in an individual patch has no bearing on that of a neighboring patch, despite the movement of individuals between the two areas. Through variation of several parameters related to disease management, we determined regions of parameter space that permit successful control. We first assumed that one population maximized the potential benefits of either (*I*) hospitalization, (*II*) hospitalization and safe burial practices, or (*III*) quarantine and safe burial practices. In contrast, the other population implemented varying levels of these management practices. We additionally considered a scenario in which populations used a combination of both hospitalization and quarantine.

We find that the positive benefits of effective intervention in one population can help overcome poor strategies in a connected population, which can lead to elimination of the pathogen on a more global scale (i.e. elimination in both populations). This holds for even low levels of movement between populations. In contrast, the same population with poor strategies would experience disease persistence in the absence of connectivity. Therefore, the interconnectedness of regions, without regard to nation boundaries and policies, is an essential consideration for a coordinated response to emerging infections.

## Theoretical Framework

We begin by introducing a transmission model of Ebola virus that ignores spatial structure but divides infectious individuals into groups based on their treatment status, allowing for variation in the infectivity arising from different treatment methods. Next, we introduce spatial structure into our model by including two populations that are connected by movement of individuals. Flexibility is embedded in the structured model to allow for potentially different treatment strategies between populations. Additionally, we derive the basic reproductive number for both the non-spatial and spatial models.

### Model without explicit space

**Figure 1:**
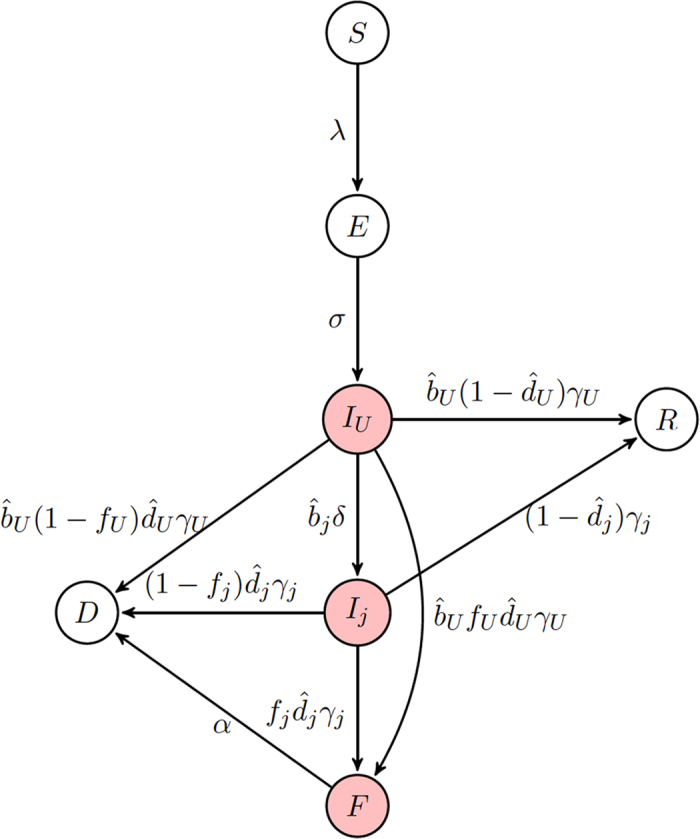
Schematic of the non-spatial model of Ebola transmission. A compartmental *SEIR*-model is used to follow Ebola transmission within a model. Here, *j* = {*Q*, *H*} for quarantined and hospitalized classes, respectively. Infectious classes are shaded.

Our initial modeling framework considers an *SEIR*-type model in the absence of explicit space (Fig. 1). We assume that living infectious individuals can fall into one of three classes: infectious but untreated (*I*_*U*_, hereafter referred to as ‘undetected’), infectious but hospitalized (*I*_*H*_), or infectious but quarantined (*I*_*Q*_). Here, quarantine is only meant to indicate the complete isolation of infected individuals, not the separation of potentially infectious individuals from the general population. We assume that disease transmission only occurs from living individuals that are either undetected or hospitalized. Additionally, transmission of Ebola may also occur from victims of disease-induced mortality. This typically occurs during funeral practices prior to burial; therefore, we create a class (*F*) comprised of deceased individuals capable of transmission. Given these different transmission routes, the overall force of infection is given by:


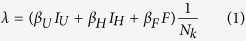


where *β*_*U*_, *β*_*H*_, and *β*_*F*_ are the *per capita* transmission rates from individuals in the undetected, hospitalized, and funeral classes, respectively. We assume that hospital treatment diminishes an individual’s overall infectivity so that *β*_*H*_ ≤ *β*_*U*_. In our model, *N*_*k*_ is the total number of individuals in the population capable of contributing to onward infection including those in the funeral class, but excluding those who are quarantined (*I*_*Q*_) and effectively removed (*D*) from the population.

Upon infection, individuals enter an exposed (*E*) class and subsequently become infectious at a rate *σ*. Under the assumption that the birth and death rates (*μ*) are equal,


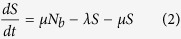



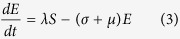


where *N*_*b*_ is the population size of individuals capable of reproduction, i.e. excluding individuals in the funeral and effectively removed classes.

After becoming infectious, individuals initially enter the undetected class and can remain in that class with probability *b*_*U*_ until leaving the class at rate *γ*_*U*_. Alternatively, individuals may subsequently enter the hospitalized or quarantined class with probabilities *b*_*H*_ and *b*_*Q*_, respectively, at a rate *δ*. These classes can be described as:


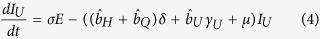



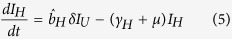



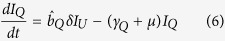


where *γ*_*H*_ and *γ*_*Q*_ are the rates at which individuals leave their respective infectious class. In order to ensure the proper exit rates from our compartments, we follow the convention of Legrand *et al*.^10^ in our equations but note that 
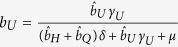
 is the fraction of exposed individuals remaining undetected, rather than 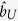. Similarly, 
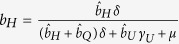
 is the fraction of individuals who are hospitalized and 
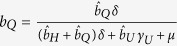
 is the fraction of individuals quarantined. Throughout, parameters with a hat appear in equations, but are not true fractions, while figure axes use the true fraction undergoing a particular intervention.

Individuals are assumed to either succumb to infection (with probability 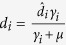, where *i* = {*U*, *H*, *Q*} is the respective infectious class) or recover (with probability 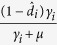). It is assumed that a fraction *f*_*i*_ (where *i* = {*U*, *H*, *Q*} is the respective infectious class) of deceased patients are provided with funerals, during which each patient remains capable of transmission. Individuals are then buried and enter an effectively removed class, *D*, in which they are no longer capable of transmission. Additionally, the remaining fraction 1 − *f*_*i*_ individuals directly enter the *D* class. We can describe these processes with the following differential equations:


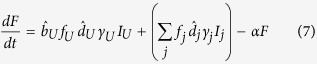



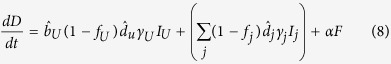



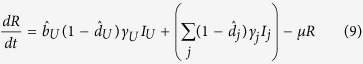


where *j* = {*H*, *Q*}. Note that 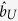 is the relative fraction of individuals that remain undetected rather than entering the hospital or quarantine, so that 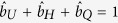.

The majority of the parameterization of our models is based upon the outbreak of Ebola virus in West Africa, beginning in late 2013 and extending into 2015. Although the incubation period varied slightly among the three countries, the measured mean incubation time across West Africa was 9–12 days^20^. The serial interval – average time from symptom onset of the index case to symptom onset of the secondary case – was consistently found to be 14–15 days^20^. As the mean time in the undetected infectious class (*γ*_*U*_) was 10 days^20^, the time spent in the hospitalized and quarantined classes was calculated to account for time first spent in the undetected class and the serial interval^20^. In other words, 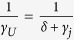. The case fatality of all three countries over the course of the epidemic was ~70%^20^ with only a minor reduction in the case fatality rate when individuals received palliative care^21^. The relative fraction of exposures that result in disease and enter a given infectious class (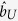, 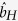, 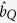) varied based on the community of interest. Values for *β*_*U*_ and *β*_*F*_ were set assuming a reproductive number *R*_0_ = 1.85, consistent with estimates of the 2013–2015 Ebola epidemic in West Africa (see Appendix A for more details). Parameters for our models can be found in Table 1.

Importantly, many of the parameters in this model vary based on policy decisions and community interventions for Ebola control. For example, the probability of remaining in the community (here, undetected) throughout the course of the infection (*b*_*U*_) increased in areas with insufficient hospital beds or health clinics. Furthermore, public health campaigns in countries affected by the epidemic highlighting the importance of careful treatment of deceased individuals as well as allotment of resources to appropriately disinfect led to safer burial practices^1^. In our model, this corresponds to increases in the probability that safe burials (1 − *f*_*j*_) are provided. We explore the benefits of such controls in the Results section.

### Basic Reproductive Number in non-spatial model

To find the basic reproductive number in the non-spatial model, we used the next generation matrix (see Appendix A for details) and obtain that


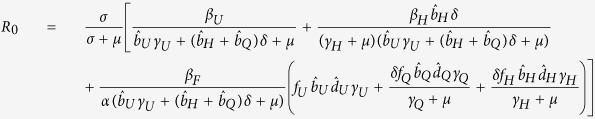


This quantity is straight forward to interpret biologically as *R*_0_ can be broken into contributions from each of the following: (i) transmission from the undetected class, divided by the exit rate from *I*_*U*_ of individuals who do not enter *I*_*H*_ or *I*_*Q*_ plus the exit rate of individuals who eventually enter *I*_*H*_ or *I*_*Q*_ (this term plays into the remaining components of *R*_0_), (ii) transmission from the individuals who have left *I*_*U*_ and enter the hospitalized class *I*_*H*_ multiplied by the mean time spent in *I*_*U*_, and (iii) transmission from individuals in the *F* class multiplied by the mean time spent in *F* and by the fraction of individuals who eventually enter the *F* class from any of the infectious classes. Each of the contributions is reduced by the loss of individuals to natural mortality before infection can be transmitted.

For an initial look at *R*_0_, we assumed that there is no control, i.e. all individuals remain in the undetected class (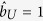 and 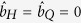), then *R*_0_ simplifies even further to:


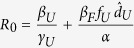


which is the transmission rate from individuals in *I*_*U*_ multiplied by the mean time spent in *I*_*U*_, added to the transmission rate from individuals who die and have a funeral multiplied by the mean time spent in *F*. We parameterize *γ*_*U*_ and *d*_*U*_ from data^20^ which leaves the remaining unknowns are: *β*_*U*_, *β*_*F*_, and *α*. The value of *α* does not have a large effect on *R*_0_, but the longer the time spent in the funeral class, the faster that *R*_0_ increases with *β*_*F*_ (Fig. 1A). For simplicity, we choose *α* = 1 for the remainder of the paper. Transmission from the undetected class (*β*_*U*_) causes faster increases in *R*_0_ relative to transmission from funerals (*β*_*F*_), due to the short amount of time in the *F* class (Fig. 1A).

### Model with explicit space

To explicitly include space in our population, we mechanistically took into account movement of subpopulations, (as in ref. 22), allowing the ability to incorporate populations of different sizes (see Appendix A for equations). Individuals are separated based on their status as home or visiting, and parameters are derived from the current local population for the individuals. Individuals leave their home population at rate *ρ* and return at rate *τ*. For consistency, there is movement between patches of susceptible, exposed, infectious and recovered individuals but not of individuals in the funeral and effectively removed class. Infection can only occur when both susceptible and infectious individuals are present in the same population.

Several parameters can differ between populations depending on their respective intervention policies. For example, case detection, hospitalization rates, and quarantine status may vary. Therefore, we allow for differences in *b*_*U*_, *b*_*H*_, and *b*_*Q*_. While we assume that the average time spent infectious (1/*γ*_*j*_) is an intrinsic property of the disease, it is possible that the probability of surviving the disease varies (i.e. 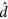
_*j*_). Burial practices may also differ between populations so that, for example, different communities are more or less likely to hold a traditional burial which leads to differences in the value of *f*_*j*_.

### Basic reproductive number in spatial model

Similar to the previous model without explicit space, we used the next generation matrix to compute the basic reproduction number numerically (see Appendix for details of *F* and *V* next generation matrices). Although we do not find an analytic form for the reproductive number due to the complexity of this model, we numerically determine *R*_0_ in our simulations.

Under simplifying assumptions, however, we recover an expression for *R*_0_ that resembles that for the non-spatial model. For example, excluding demographics (*μ* = 0) and intervention (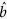
_*H*1_ = 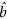
_*H*2_ = 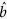
_*Q*1_ = 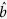
_*Q*2_ = 0), assuming equal movement (*ρ* = *τ*) and equal population sizes, and all other parameters equal between the two populations (i.e. *β*_*U*1_ = *β*_*U*2_, *β*_*F*1_ = *β*_*F*2_), the basic reproduction number becomes: 
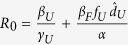
.

Relaxing these assumptions such that the populations are no longer identical (i.e. *β*_*U*1_ ≠ *β*_*U*2_, *β*_*F*1_ ≠ *β*_*F*2_) but still excluding movement (*τ* = *ρ* = 0), then the next generation matrix has two non-zero eigenvalues:


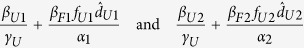


the larger of which is the value for *R*_0_. Indeed, in the absence of movement (*τ* = *ρ* = 0) the two populations act independently and the analysis of the non-spatial model is recovered for each subpopulation. Importantly, the value for *R*_0_ directly depends on both populations. This paints a more global picture of the overall success of the Ebola epidemic.

## Results

We consider four strategies for disease intervention. Given that one population has optimally implemented an intervention strategy (hereafter referred to as “population 1”), we determine implications for control of an epidemic when a second population (hereafter referred to as “population 2”) has sub-optimal management in the presence of movement between populations. In the final strategy, we investigate two interventions employed at varying levels in the same population. When referring to a parameter specific to a particular population, we will include a second index in the subscript to identify whether we are referring to population 1 or 2. The methods we consider are:

- *Hospitalization*. We assume that population 1 successfully hospitalizes all infectious individuals as we as eliminated hospital transmission (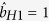, *b*_*H*1_ ≈ 1, *β*_*H*1_ = 0). In contrast, population 2 has varying levels of hospitalization (*b*_*H*_) and transmission within hospitals (*β*_*H*_).
- *Hospitalization and safe burial practices*. Population 1 is as in method (I), with the addition that all hospital deaths are treated with safe burials (*f*_*H*1_ = 0). We assume that all cases are hospitalized in population 2 (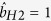, *b*_*H*2_ ≈ 1), but it varies in its ability to eliminate transmission within hospitals (*β*_*H*2_) as well as its use of safe burial practices following death after hospitalization (*f*_*H*2_).
- *Quarantine and safe burial practices*. We assume that population 1 successfully quarantines all infected individuals (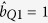, *b*_*Q*1_ ≈ 1) and that all deaths are treated with safe burials (*f*_*Q*1_ = 0). In contrast, population 2 has varying levels of quarantine (*b*_*Q*2_) and usage of safe burial practices (*f*_*Q*2_).
- *Hospitalization and quarantine*. In this final scenario, we assume that population 1 has optimally implemented a single intervention strategy but that population 2 uses a mixed strategy implementing both hospitalization and quarantine with varying degrees of success (*b*_*H*2_ and *b*_*Q*2_).

To be consistent with the 2013–2015 West African epidemic, we assume that *R*_0_ = 1.85^20^. However, the relative magnitudes of the transmission rate of individuals with undetected infections (*β*_*U*_) and the transmission rate during traditional funerals (*β*_*F*_) is not well known. We therefore obtain a more global picture of the dynamics by varying the values of *β*_*U*_ and *β*_*F*_ while maintaining the value *R*_0_ = 1.85 in the absence of intervention strategies. To assess when an the epidemic can be prevented for each of the above strategies, we then identify regions of parameter space where the intervention strategy forces *R*_0_ < 1.

### Hospitalization

**Figure 2:**
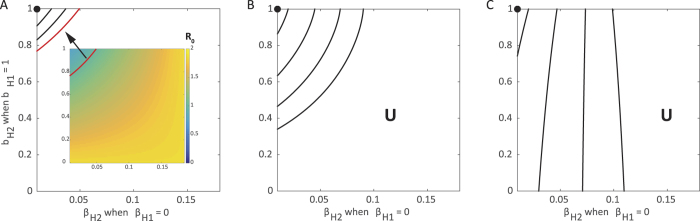
Control of the epidemic through hospitalization under varying movement between subpopulations. Each contour corresponds to the combinations of *β*_*H*2_ and *b*_*H*2_ where *R*_0_ = 1 when population 1 achieves full hospitalization (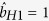, *b*_*H*1_ ≈ 1) allowing no onward transmission in the hospital (*β*_*H*1_ = 0) considering varying levels of movement: (**A**) none, (**B**) low (*τ* = *ρ* = 0.05), and (**C**) high (*τ* = *ρ* = 500). The red highlighted contour in (**A**) is equivalent to the solid red line in the inset. Each line represents a particular choice of *β*_*U*_ and *β*_*F*_ such that *R*_0_ = 1.85 when no control measures are introduced. Above and to the left of contours *R*_0_ < 1 and the epidemic can be controlled. While below and to the right, the epidemic can never be controlled and *R*_0_ > 1 (**U** = uncontrolled area), as illustrated by values of *R*_0_ in the inset of (**A**). Standard values are used for other parameters as indicated in Table 1. For reference, the filled black circles indicate where both populations implement identical controls: *b*_*H*_ = 1, *β*_*H*_ = 0.

Assuming that population 1 has achieved complete hospitalization without the possibility of further transmission, we determine the plausibility of control when population 2 varies in its ability to detect and hospitalize infected individuals (*b*_*H*2_) as well as the transmission rate in the hospital (*β*_*H*2_). The relative balance of transmission for the hospitalized class and the funeral class will depend on the transmission rate from each of these classes, *β*_*H*2_ and *β*_*F*2_, respectively. *R*_0_ < 1 can only be achieved if transmission from *I*_*H*_ individuals is low and a substantial portion of the population enters the hospital (above and to the left of the red curve corresponding to *R*_0_ = 1 in Fig. 2A inset).

Over a range of combinations of *β*_*U*_ and *β*_*F*_, all corresponding to *R*_0_ = 1.85, we find when hospitalization is able to control the epidemic, i.e. the contour corresponding to *R*_0_ = 1 (Fig. 2). Here, control is only possible if *I*_*U*_ transmission is high relative to *F* transmission, *β*_*H*_ is relatively small, and *b*_*H*_ is high (Fig. 2 upper left corner). In other words, assuming that transmission within hospitals is small, increasing hospitalization rates can have the most positive effect when the majority of transmission is driven by undetected cases rather than unsafe burial practices.

Interestingly, when there exists no movement between the subpopulations, the region where the epidemic is controllable is smallest (Fig. 2A), as no benefit is accrued from movement of individuals from the region where hospitalization is ubiquitous. Thus, a larger proportion of the population needs to enter the hospital for control to be achieved. As movement between the two population increases, the controllable region grows, particularly for increases in the hospital transmission rate (*β*_*H*2_) (Fig. 2B,C).

### Hospitalization and safe burial practices

**Figure 3:**
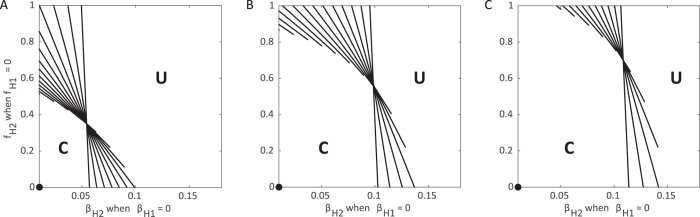
Control of the epidemic through hospitalization and safe burial practices under varying movement between subpopulations. Each contour corresponds to the combinations of *β*_*H*2_ and *f*_*H*2_ where *R*_0_ = 1 assuming maximized hospitalization (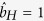, *b*_*H*_ ≈ 1) in both populations while population 1 has no funerals (*f*_*H*1_ = 0) and no transmission from the hospitalized class (*β*_*H*1_ = 0) considering varying levels of movement: **(A)** none, **(B)** low (*τ* = *ρ* = 0.05), and **(C)** high (*τ* = *ρ* = 500). Each line represents a particular choice of *β*_*U*_ and *β*_*F*_ such that *R*_0_ = 1.85 when no control measures are introduced. Above and to the right of contours *R*_0_ > 1 and the epidemic can never be controlled (**U** = uncontrolled area), while below and to the left, the epidemic can be controlled and *R*_0_ < 1 (**C** = controlled area). Standard values are used for other parameters as indicated in Table 1. The contours end prior to reaching the axes to ensure *β*_*H*_ ≤ *β*_*U*_. For reference, the filled black circles indicate where both populations implement identical controls: *β*_*H*_ = 0, *f*_*H*_ = 0.

The second method for control is similar to the first, but additionally assumes that all hospital deaths in population 1 are treated with safe burials. Assuming that population 2 is now successful in hospitalizing all cases (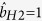, *b*_*H*2_ ≈ 1), we vary its ability to reduce transmission within hospitals (*β*_*H*2_) as well as the fraction of individuals having a traditional burial following death within a hospital (*f*_*H*2_). We find there is a linear relationship between *β*_*H*2_ and *f*_*H*2_ (i.e. the contour corresponding to *R*_0_ = 1 is a line). When *f*_*H*2_ and *β*_*H*2_ are both low, below and to the left of lines in Fig. 3, the epidemic is controllable as it is possible to force *R*_0_ < 1 through hospitalizations.

We find this line for various relative contributions of transmission from *I*_*U*_ and *F*, with contributions from each denoted 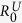 and 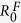, respectively (see details in Appendix A):


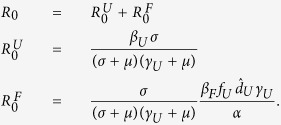


For intermediate values of *β*_*H*2_ and *f*_*H*2_ the ability to control the epidemics differs on whether *β*_*H*2_ is high and *f*_*H*2_ is low, or vice versa. When *β*_*H*2_ is high *f*_*H*2_ is low, transmission is high in hospitals but few individuals who die have unsafe burials. Here, *R*_0_ can only be brought below one by reducing the number of individuals who have unsafe burials. Control is more feasible if transmission at funerals is the main contributor to *R*_0_, rather than undetected cases (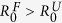). In other words, even when *F* transmission is high, reducing the number of individuals receiving an unsafe burial (low *f*_*H*2_) can still bring *R*_0_ below one.

Contrarily, when *β*_*H*2_ is low and *f*_*H*2_ is high, transmission is low in hospitals but most individuals receive a safe funeral. As transmission in the hospital class is reduced, more individuals who die following hospitalization can have an unsafe burial and still force *R*_0_ below one. Control is more feasible if transmission from undetected cases is the primary contributor to *R*_0_, rather than cases originating from unsafe burials (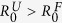). Higher values of *β*_*U*_ correspond to minimal transmission during funerals, and therefore, more individuals can have unsafe burials and *R*_0_ can still be forced to be below one.

Similar to the previous section, the amount of movement between the populations contributes to the ability for the total population to control the epidemic (Fig. 3B,C). The control available in one subpopulation assists the lower level of control introduced in the other and has a greater impact as the movement between the two groups increases.

### Quarantine and safe burial practices

**Figure 4:**
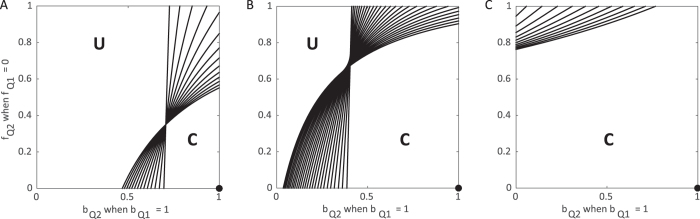
Control of the epidemic through quarantine under varying movement between subpopulations. Each contour corresponds to the combinations of *b*_*Q*2_ and *f*_*Q*2_ where *R*_0_ = 1 assuming all infectious individuals in population 1 are in quarantine (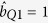, *b*_*Q*1_ ≈ 1) and none receive funerals (*f*_*Q*1_ = 0) considering varying levels of movement: (**A**) none, (**B**) low (*τ* = *ρ* = 0.05), and (**C**) high (*τ* = *ρ* = 500). Each line represents a particular choice of *β*_*U*_ and *β*_*F*_ such that *R*_0_ = 1.85 when no control measures are introduced. Above and to the left of contours *R*_0_ > 1 and the epidemic can never be controlled (**U** = uncontrolled area), while below and to the right, the epidemic can be controlled and *R*_0_ < 1 (**C** = controlled area). Standard values are used for other parameters as indicated in Table 1. The contours end before the axes to ensure *β*_*H*_ ≤ *β*_*U*_. For reference, the filled black circles indicate where both populations implement identical controls: *b*_*Q*_ = 1, *f*_*Q*_ = 0.

In the third method for control, we assume that population 1 quarantines all infected individuals and that all deaths following quarantine are treated with safe burials. We first look at the effect of varying *b*_*Q*2_ (fraction of individuals who enter the *Q* class) and *f*_*Q*2_ (fraction of individuals who have a traditional funeral following disease-induced death in the *Q* class). In this case, there is no need to vary the infectivity of individuals in *I*_*Q*_ as they never contribute to infections, i.e. *β*_*Q*_ = 0. Here, if there is little transmission from *F* and high transmission from *I*_*U*_ individuals, then the system is controllable as long as enough individuals enter the *Q* class (Fig. 4A). However, if transmission during *F* is at an intermediate or high level, then it is not possible to control if there are a large number of quarantined individuals still having funerals (large *f*_*Q*_).

For intermediate values of *b*_*Q*2_ and *f*_*Q*2_ the ability to control the epidemic depends on whether both *b*_*Q*2_ and *f*_*Q*2_ are high, or both are low. When *b*_*Q*2_ is high, the proportion of individuals progressing to funerals (*f*_*Q*2_) can be relatively high, even above 50%, and *R*_0_ can be brought below one. As there is no transmission in the *I*_*Q*_ class, funerals resulting from this class are the primary contribution to onward infections. Indeed, if *b*_*Q*2_ is low, so most infected individuals remain undetected, regardless of the those safely buried following quarantine, the epidemic is never controllable (Fig. 4). Contrarily, when *b*_*Q*2_ is low, some individuals must receive a funeral for control to be achieved.

As quarantine, by definition, limits transmission (*β*_*Q*_ = 0), when population 1 achieves safe burial practices (*f*_*Q*1_ = 0), the ability to control is greatly affected by the amount of movement between populations (Fig. 4B,C). At lower levels of movement, the transition of an epidemic from uncontrollable to controllable is more dependent on the proportion of the population entering quarantine (Fig. 4B). With a high level of movement, even a minor reduction in the proportion of individuals receiving unsafe burials for a small proportion of the second population leads to control the epidemic (Fig. 4C).

### Combining hospitalization and quarantine

**Figure 5:**
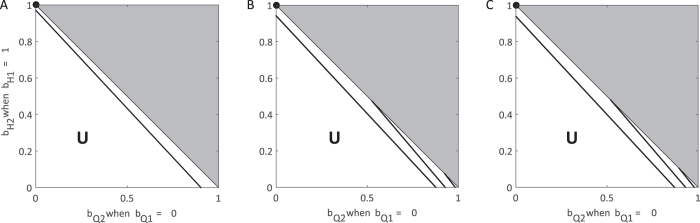
Control of the epidemic using a combination of hospitalization and quarantine in one subpopulation and only hospitalization in the other. Each contour corresponds to the combinations of *b*_*Q*_ and *b*_*H*_ in subpopulation 2 where *R*_0_ = 1 assuming all infectious individuals are hospitalized in the subpopulation 1 (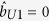, 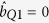, 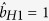) considering varying levels of movement: (**A**) none, (**B**) low (*τ* = *ρ* = 0.05), and (**C**) high (*τ* = *ρ* = 500). Each line represents a particular choice of *β*_*U*_ and *β*_*F*_ such that *R*_0_ = 1.85 when no control measures are introduced. To the left of contours *R*_0_ > 1 and the epidemic can never be controlled (U = uncontrollable area). Standard values are used for other parameters as indicated in Table 1 except unsafe burials which occur with 50% probability (*f*_*j*_ = 0.5).

**Figure 6:**
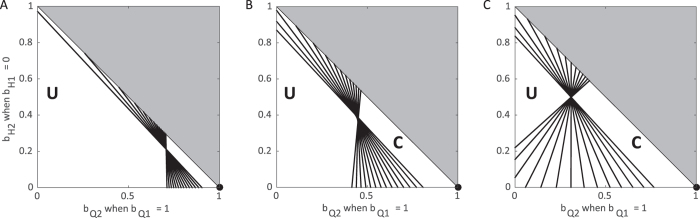
Control of the epidemic using a combination of hospitalization and quarantine in one subpopulation and only quarantine in the other. Each contour corresponds to the combinations of *b*_*Q*_ and *b*_*H*_ in subpopulation 2 where *R*_0_ = 1 assuming all infectious individuals are quarantined in the subpopulation 1 (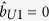, 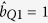, 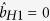) considering varying levels of movement: (**A**) none, (**B**) low (*τ* = *ρ* = 0.05), and (**C**) high (*τ* = *ρ* = 500). Each line represents a particular choice of *β*_*U*_ and *β*_*F*_ such that *R*_0_ = 1.85 when no control measures are introduced. To the left of contours *R*_0_ > 1 and the epidemic can never be controlled (U = uncontrollable area), while to the right of contours the epidemic is controllable (C = controllable area). Standard values are used for other parameters as indicated in Table 1 except unsafe burials which occur with 50% probability (*f*_*j*_ = 0.5).

In contrast to our examination of the use of only hospitalization or only quarantine, populations have the opportunity to apply hospitalization and quarantine in combination, typically with some proportion of the population remaining undetected. When two populations are linked through movement of individuals, the use of both hospitalization and quarantine leads to control of the epidemic more often than a single intervention alone (Figs 5 and 6). When one subpopulation chooses to apply a single intervention, a combination intervention strategy in the other population can lead to control the epidemic (Fig. 5). When quarantine is chosen as the sole strategy, the total population will have a controllable epidemic for a wider range of mixing since there is no onward transmission from the quarantined population (*β*_*Q*_ = 0) (Fig. 6).

## Discussion

Several countries in West Africa, in particular Liberia, Sierra Leone and Guinea, experienced significant morbidity and mortality during the Ebola epidemic from 2013–2015. At the time of this epidemic there was no known vaccine or drug, so effective disease control required coordinated efforts that include both standard strategies, such as hospitalization, as well as community efforts, such as safe burial practices. Not only are such efforts difficult to implement in practice, but there is also added complexity with connectivity between populations that have different policies in place, as was the case in these three countries. In this paper, we explore the role of movement between two theoretical populations in the overall success of practices designed to minimize the extent of Ebola epidemics.

We used the basic reproductive number, *R*_0_, as a metric for evaluating the overall success of a management strategy. In our simulations, we considered two populations with varying management practices and then determined when control is possible as movement between spatial locations increases. We first assumed that one population maximized the potential benefits of either (*I*) hospitalization, (*II*) both hospitalization and safe burial practices, or (*III*) quarantine and safe burial practices. In contrast, the other population implemented varying levels of these management practices. We additionally considered one final scenario in which one populations used a combination of hospitalization and quarantine.

Importantly, in all scenarios we showed, a control strategy that is effective (*R*_0_ < 1) in a spatially isolated population may no longer be effective in the presence of connectivity with another population that has weaker management policies in place. This results from deriving a more global value of *R*_0_ that accounts for interconnected populations that are simultaneously experiencing Ebola outbreaks; in the presence of space, *R*_0_ depends on the dynamics within each spatial location as well as on interactions between them. Interestingly, the consequences of connectivity is most substantial when movement rates are relatively small. Here, while local control in one population may be independently effective, the more global picture demonstrates that the epidemic will continue to progress. In contrast, Ebola management is possible under a wider range of implementation strategies for the second population as movement rates increase. This is because the total population becomes more homogeneous, and the second population can benefit directly from the first population’s policies.

In general, the overall success of each management scenario depends on the associated levels of control in the second population. In our model quarantine is more effective than hospitalization because of the differences in transmission rates from individuals in the hospitalized as compared to the quarantine class; while transmission is lower in hospitals as compared to undetected individuals, transmission is eliminated from individuals in quarantine. In practice, however, quarantine may not completely abrogate transmission as both infected and uninfected individuals may be forced together. While hospitalization is necessary to minimize the morbidity and mortality associated with infected patients, hospitals must take measures to minimize the transmission from individuals infected with Ebola to health care workers and other patients in the hospital.

Our results highlight the direct benefit of safe burial practices on the controllability of Ebola epidemics. This is most evident in Fig. 5; including safe burial practices in addition to a combination of hospitalization and quarantine greatly increases the range of successful management implementations. In fact, when all Ebola-related deaths are provided traditional burials, Ebola epidemics are almost never controllable. Analysis of previous epidemics has also indicated the importance of transmission during traditional funeral practices^23^. As a result, proper safe burial has become an important tenant of Ebola control^4^,^5^,^6^.

We evaluated the potential success of several controls designed to combat Ebola when policies differ between two distinct populations. Our findings indicate that the effectiveness of policies for Ebola management can very dramatically depending on how connected a given location is to neighboring regions. The potential imbalance of resources could impact even the populations receiving the bulk of resources, if the populations are interconnected. As the worldwide travel and porous borders are common, no population can consider itself in isolation. More generally, this study highlights the critical importance of considering spatial structure when evaluating disease management strategies.

## Additional Information

**How to cite this article**: Blackwood, J. C. and Childs, L. M. The role of interconnectivity in control of an Ebola epidemic. *Sci. Rep*. **6**, 29262; doi: 10.1038/srep29262 (2016).

## Supplementary Material

Supplementary Information

## Figures and Tables

**Table 1:** Model parameters.

Symbol	Description	Value	Range	Source
*β*_*U*_	Transmission rate from undetected cases	0.0969	[0, 0.184]	see text
*β*_*F*_	Transmission rate from funerals	1.26	[0, 2.502]	see text
*b*_*j*_	Fraction of exposures that enter infectious class *I*_*j*_	1	[0, 1]	varies
1/*σ*	Mean time spent in the *E* class	10d	–	20
1/*γ*_*U*_	Mean time spent in infectious class *I*_*U*_	10d	–	20
1/*δ*	Mean time spent undetected	3.5d	–	20
1/*γ*_*j*_	Mean time spent in infectious class *I*_*j*_, *j* = {*H*, *Q*}	6.5d	–	see text
*d*_*j*_	Fraction of individuals in class *I*_*j*_ who die	0.7	–	20
*f*_*j*_	Fraction of deceased given an unsafe funeral	1	[0, 1]	varies
1/*α*	Mean time spent in the funeral class	1d	[1, 5]d	10
1/*μ*	*per capita* death rate	55y	46–62y	24
1/*ρ*	Movement from home population	0	[0, 500]	varies
1/*τ*	Movement into home population	0	[0, 500]	varies
