# Supplementary material for: The role of interconnectivity in control of an Ebola epidemic
Source: Sci Rep. 2016 Jul 7;6:29262. doi: 10.1038/srep29262 (PMC4935855; doi:10.1038/srep29262)
Supplement: Supplementary Information [file srep29262-s1.pdf]

The role of interconnectivity in control of an Ebola epidemic

JC Blackwood[1,†], LM Childs[1,2]

[1]Department of Mathematics and Statistics
Williams College, Williamstown, MA 01267, USA

[2]Center for Communicable Disease Dynamics
Harvard T.H. Chan School of Public Health, Boston, MA 02115, USA

[†]*Both authors contributed equally to this work*

Electronic Supplementary Materials

Contents

A Online Appendix: Model Details **2**

A.1 Parameterization 2

A.2 Derivation of R_0: non-spatial model 2

A.3 Model with spatial coupling 3

A.4 Derivation of R_0: spatial model 7

A.5 Control by hospital care: linear relationship between f_H and β_H 9

A Online Appendix: Model Details

A.1 Parameterization

As stated in the main text we choose an R_0 value of 1.85, which is consistent with estimates of the 2013–2015 West Africa Ebola epidemic **?**. We assume that the estimate of R_0 is based on transmission in the absence of hospital and community interventions. Therefore, we select values of β_H and β_F for which $R_0 = 1.85$ holds without intervention, i.e. $\hat{b}_H = \hat{b}_Q = 0$ and $\hat{b}_U = 1$. This is captured by the white line in Figure 1. For all simulations, the various curves correspond to different relative contributions of transmission from funerals and from undetected individuals to the overall R_0. All other model parameters are provided in Table 1.

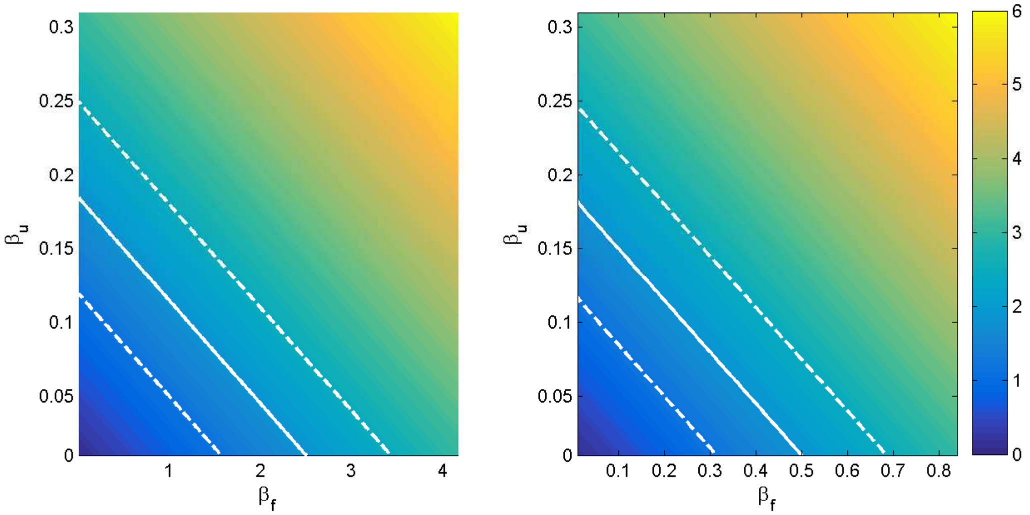

Figure 1. The value of R_0 as β_F and β_U are varied, assuming no intervention ($\hat{b}_H = \hat{b}_Q = 0$, $\hat{b}_U = 1$) and the funeral class lasts either**(A)** 1 day ($\alpha = 1$) or **(B)** 5 days ($\alpha = 5$). The solid white line captures all combinations that have a corresponding $R_0 = 1.85$. The dashed lines display values of 1.2 and 2.5, which were the lowest and highest values provided in **?**. Note the x-scale difference in **(A)** and **(B)**.

A.2 Derivation of R_0: non-spatial model

Here, we derive the basic reproductive number, R_0, for the non-spatial model using the next generation matrix method. The relevant classes are E, I_U, I_H, I_Q, and F. Note that although I_Q does not contribute to the force of infection, it is the source of some new

individuals into the F class. We only include new infections in, F, the "gains" matrix, which is given by:

$$F_{ij} = \begin{cases} \dfrac{\beta_U S}{N_k} & \text{if } i = 1, j = 2 \\ \dfrac{\beta_H S}{N_k} & \text{if } i = 1, j = 3 \\ \dfrac{\beta_F S}{N_k} & \text{if } i = 1, j = 5 \\ 0 & \text{otherwise} \end{cases}$$

Next, V is the "loss" matrix, and it is given by:

$$V = \begin{bmatrix} \sigma + \mu & 0 & 0 & 0 & 0 \\ -\sigma & (\hat{b}_H + \hat{b}_Q)\delta + \hat{b}_U\gamma_U + \mu & 0 & 0 & 0 \\ 0 & -\hat{b}_H\delta & \gamma_H + \mu & 0 & 0 \\ 0 & -\hat{b}_Q\delta & 0 & \gamma_Q + \mu & 0 \\ 0 & -\hat{b}_U f_U \hat{d}_U \gamma_U & -f_H \hat{d}_H \gamma_H & -f_Q \hat{d}_Q \gamma_Q & \alpha \end{bmatrix}$$

R_0 is the spectral radius of FV^{-1}, and it is given by

$$\begin{aligned} R_0 &= \frac{\sigma}{\sigma + \mu}\Bigg[\frac{\beta_U}{\hat{b}_U\gamma_U + (\hat{b}_H + \hat{b}_Q)\delta + \mu} \\ &+ \frac{\beta_H \hat{b}_H \delta}{(\gamma_H + \mu)\left(\hat{b}_U\gamma_U + (\hat{b}_H + \hat{b}_Q)\delta + \mu\right)} \\ &+ \frac{\beta_F}{\alpha\left(\hat{b}_U\gamma_U + (\hat{b}_H + \hat{b}_Q)\delta + \mu\right)}\left(f_U\hat{b}_U\hat{d}_U\gamma_U + \frac{\delta f_Q \hat{b}_Q \hat{d}_Q \gamma_Q}{\gamma_Q + \mu} + \frac{\delta f_H \hat{b}_H \hat{d}_H \gamma_H}{\gamma_H + \mu}\right)\Bigg]. \end{aligned}$$

A full description of the interpretation of R_0 is included in the main text.

A.3 Model with spatial coupling

We extend our model to account for migration between two distinct subpopulations. To do this mechanistically, we explicitly take into account the migrating subpopulations, thus allowing the ability to incorporate populations of different sizes **?**. Every individual has a home population (denoted by superscript x or y), and there is migration between populations of all living individuals (susceptible, exposed, infectious and recovered). We assume there is no migration of individuals in the funeral F and effectively removed D class. Individuals leave their home population at rate ρ and return at rate τ. Infectious

individuals may be too sick to migrate and have slightly reduced migration parameters, $\hat{\rho} \leq \rho$ and $\hat{\tau} \leq \tau$, although for simplicity we typically assume $\rho = \hat{\rho}$ and $\tau = \hat{\tau}$. Infection can only occur when both susceptible and infectious individuals are present in the same subpopulation which results in the following force of infection:

$$\lambda_i = \left(\beta_{Ui}(I_U^{ii} + I_U^{ji}) + \beta_{Hi}(I_H^{ii} + I_H^{ji}) + \beta_{Fi}F^i\right)\frac{1}{N_I^i}$$

For each of the classes the first superscript refers to the home population and the second refers to the population the individuals are currently situated in (i.e., I^{ji} indicates infectious individuals who live in population j that are currently in population i). Parameters have subscripts denoting the population they are associated with. Here, transmission rate β will correspond to the population currently situated in. The population N_I^i is composed of individuals currently situated in population i who are involved in transmission (S, E, I_U, I_H, F, and R). Here, I_Q is excluded from N_I because individuals do not contribute to the force of infection.

Using the non-spatial model as a baseline, the dynamics of the populations follow a similar pattern but with the inclusion of migration between populations (the final two terms in each equation below). In the home population, susceptible individuals are born into the population from individuals only in their subpopulation $N_B^{xx} = S^{xx} + E^{xx} + \sum_j I_j^{xx} + R^{xx}$:

$$\frac{dS^{xx}}{dt} = \mu N_B^{xx} - \lambda_x S^{xx} - \mu S^{xx} + \tau S^{xy} - \rho S^{xx}$$

At rate λ_x the susceptible individuals in S^{xx} enter the exposed class E^{xx} and become infectious at rate σ:

$$\frac{dE^{xx}}{dt} = \lambda_x S^{xx} - (\sigma + \mu)E^{xx} + \tau E^{xy} - \rho E^{xx}$$

After becoming infectious, individuals enter the undetected class and either remain there were probability $\hat{b}_{Ux}$ or enter the hospital ($\hat{b}_{Qx}$) or quarantine ($\hat{b}_{Qx}$):

$$\begin{aligned}
\frac{dI_U^{xx}}{dt} &= \sigma E^{xx} - \left((\hat{b}_{Hx} + \hat{b}_{Qx})\delta + \hat{b}_{Ux}\gamma_U + \mu\right) I_U^{xx} + \hat{\tau} I_U^{xy} - \hat{\rho} I_U^{xx} \\
\frac{dI_H^{xx}}{dt} &= \hat{b}_{Hx}\delta I_U^{xx} - (\gamma_H + \mu)\, I_H^{xx} + \hat{\tau} I_H^{xy} - \hat{\rho} I_H^{xx} \\
\frac{dI_Q^{xx}}{dt} &= \hat{b}_{Qx}\delta I_U^{xx} - (\gamma_Q + \mu)\, I_Q^{xx} + \hat{\tau} I_Q^{xy} - \hat{\rho} I_Q^{xx}
\end{aligned}$$

where γ_H, γ_Q are the rates at which individuals leave their respective infective class. These rates are identical irrespective of the population.

Analogously to the non-spatial model, individuals leaving the infectious classes are assumed to either succumb to infection (with probability $\hat{d}_j$, where $j = \{H, Q\}$ is the respective infective class) or recover (with probability $1 - \hat{d}_j$). It is assumed that a fraction f_j of deceased patients are provided with funerals, during which each patient remains capable of transmission. Individuals are then buried after α days and enter a effectively removed class, D, in which they are no longer capable of transmission. Additionally, the remaining fraction $1 - f_j$ individuals directly enter the D class. We can describe these processes with the following differential equations:

$$\begin{aligned}
\frac{dF^x}{dt} &= \hat{b}_{Ux} f_{Ux} \hat{d}_{Ux} \gamma_U (I_j^{xx} + I_j^{yx}) + \left(\sum_j f_{jx} \hat{d}_{jx} \gamma_j (I_j^{xx} + I_j^{yx}) \right) - \alpha_x F^x \\
\frac{dD^x}{dt} &= \hat{b}_{Ux}(1 - f_{Ux}) \hat{d}_{Ux} \gamma_U (I_j^{xx} + I_j^{yx}) + \left(\sum_j (1 - f_{jx}) \hat{d}_{jx} \gamma_j (I_j^{xx} + I_j^{yx}) \right) + \alpha_x F^x \\
\frac{dR^{xx}}{dt} &= \hat{b}_{Ux}(1 - \hat{d}_{Ux}) \gamma_U (I_U^{xx} + I_U^{yx}) + \left(\sum_j (1 - \hat{d}_{jx}) \gamma_j I_j^{xx} \right) - \mu R^{xx} + \tau R^{xy} - \rho R^{xx}
\end{aligned}$$

where $j =$ H, Q. As we assume, deceased individuals do not migrate, there is only a single home population for F and D.

Upon migrating to the other population, individuals enter temporary populations which follow similar dynamics to the home populations:

$$\begin{aligned}
\frac{dS^{xy}}{dt} &= \mu N_B^{xy} - \lambda_y S^{xy} - \mu S^{xy} - \tau S^{xy} + \rho S^{xx} \\
\frac{dE^{xy}}{dt} &= \lambda_y S^{xy} - (\sigma + \mu) E^{xy} - \tau E^{xy} + \rho E^{xx} \\
\frac{dI_U^{xy}}{dt} &= \sigma E^{xy} - \left((\hat{b}_{Hy} + \hat{b}_{Qy}) \delta + \hat{b}_{Uy} \gamma_U + \mu \right) I_U^{xy} - \hat{\tau} I_U^{xy} + \hat{\rho} I_U^{xx} \\
\frac{dI_H^{xy}}{dt} &= \hat{b}_{Hy} \delta I_U^{xy} - (\gamma_H + \mu) I_H^{xy} - \hat{\tau} I_H^{xy} + \hat{\rho} I_H^{xx} \\
\frac{dI_Q^{xy}}{dt} &= \hat{b}_{Qy} \delta I_U^{xy} - (\gamma_Q + \mu) I_Q^{xy} - \hat{\tau} I_Q^{xy} + \hat{\rho} I_Q^{xx} \\
\frac{dR^{xy}}{dt} &= \hat{b}_{Uy}(1 - \hat{d}_{Uy}) \gamma_U I_U^{xy} + \left(\sum_j (1 - \hat{d}_{jy}) \gamma_j I_j^{xy} \right) - \mu R^{xy} - \tau R^{xy} + \rho R^{xx}
\end{aligned}$$

where $x, y = 1, 2, x \neq y$ and $j =$ H, Q.

Parameters are derived from the population where the individuals is currently situated and can differ between subpopulations: $\hat{b}_j, \hat{d}_j$ (different practices for how cases are

handled) and α_j, f_j (different practices in how burials are handled). Parameters characteristic of the disease, such as γ_j and σ, do not change between populations. All parameters can be found in Table 1.

A.4 Derivation of R_0: spatial model

We used the next generation matrix to compute the basic reproductive number, R_0, for the spatially coupled model. We denote the fraction of the susceptible population in population i as S^i, such that $S^1 + S^2 = 1$. Similar to the non-spatial model, the relevant classes are E^i, I_U^i, I_H^i, I_Q^i, and F^j, but now there is a specific classes for each subpopulation ($i = \{xx, xy, yx, yy\}$, $j = \{x, y\}$). Thus, we find our "gains" matrix to be:

$$F = \begin{bmatrix}
0 & \beta_{U1}S^1 & \beta_{H1}S^1 & 0 & \beta_{F1}S^1 & 0 & 0 & 0 & 0 & 0 & 0 & 0 & 0 & 0 & 0 & \beta_{U1}S^1 & \beta_{H1}S^1 & 0 \\
0 & 0 & 0 & 0 & 0 & 0 & 0 & 0 & 0 & 0 & 0 & 0 & 0 & 0 & 0 & 0 & 0 & 0 \\
0 & 0 & 0 & 0 & 0 & 0 & 0 & 0 & 0 & 0 & 0 & 0 & 0 & 0 & 0 & 0 & 0 & 0 \\
0 & 0 & 0 & 0 & 0 & 0 & 0 & 0 & 0 & 0 & 0 & 0 & 0 & 0 & 0 & 0 & 0 & 0 \\
0 & 0 & 0 & 0 & 0 & 0 & 0 & 0 & 0 & 0 & 0 & 0 & 0 & 0 & 0 & 0 & 0 & 0 \\
0 & 0 & 0 & 0 & 0 & 0 & \beta_{U2}S^1 & \beta_{H2}S^1 & 0 & \beta_{F2}S^1 & 0 & \beta_{U2}S^1 & \beta_{H2}S^1 & 0 & 0 & 0 & 0 & 0 \\
0 & 0 & 0 & 0 & 0 & 0 & 0 & 0 & 0 & 0 & 0 & 0 & 0 & 0 & 0 & 0 & 0 & 0 \\
0 & 0 & 0 & 0 & 0 & 0 & 0 & 0 & 0 & 0 & 0 & 0 & 0 & 0 & 0 & 0 & 0 & 0 \\
0 & 0 & 0 & 0 & 0 & 0 & 0 & 0 & 0 & 0 & 0 & 0 & 0 & 0 & 0 & 0 & 0 & 0 \\
0 & 0 & 0 & 0 & 0 & 0 & 0 & 0 & 0 & 0 & 0 & 0 & 0 & 0 & 0 & 0 & 0 & 0 \\
0 & 0 & 0 & 0 & 0 & 0 & \beta_{U2}S^2 & \beta_{H2}S^2 & 0 & \beta_{F2}S^2 & 0 & \beta_{U2}S^2 & \beta_{H2}S^2 & 0 & 0 & 0 & 0 & 0 \\
0 & 0 & 0 & 0 & 0 & 0 & 0 & 0 & 0 & 0 & 0 & 0 & 0 & 0 & 0 & 0 & 0 & 0 \\
0 & 0 & 0 & 0 & 0 & 0 & 0 & 0 & 0 & 0 & 0 & 0 & 0 & 0 & 0 & 0 & 0 & 0 \\
0 & 0 & 0 & 0 & 0 & 0 & 0 & 0 & 0 & 0 & 0 & 0 & 0 & 0 & 0 & 0 & 0 & 0 \\
0 & \beta_{U1}S^2 & \beta_{H1}S^2 & 0 & \beta_{F1}S^2 & 0 & 0 & 0 & 0 & 0 & 0 & 0 & 0 & 0 & 0 & \beta_{U1}S^2 & \beta_{H1}S^2 & 0 \\
0 & 0 & 0 & 0 & 0 & 0 & 0 & 0 & 0 & 0 & 0 & 0 & 0 & 0 & 0 & 0 & 0 & 0 \\
0 & 0 & 0 & 0 & 0 & 0 & 0 & 0 & 0 & 0 & 0 & 0 & 0 & 0 & 0 & 0 & 0 & 0 \\
0 & 0 & 0 & 0 & 0 & 0 & 0 & 0 & 0 & 0 & 0 & 0 & 0 & 0 & 0 & 0 & 0 & 0 \\
0 & 0 & 0 & 0 & 0 & 0 & 0 & 0 & 0 & 0 & 0 & 0 & 0 & 0 & 0 & 0 & 0 & 0
\end{bmatrix}$$

The gains matrix is completely independent of migration into or out of the population but does include the fraction of the susceptible population in each population. This term does not appear in the "loss" matrix, V, which is given by:

$$V = \begin{bmatrix}
\sigma+\mu+\rho & 0 & 0 & 0 & 0 & 0 & 0 & 0 & 0 & 0 & -\hat{\tau} & 0 & 0 & 0 & 0 & 0 & 0 & 0 \\
-\sigma & (\hat{b}_{H1}+\hat{b}_{Q1})\delta+\hat{b}_{U1}\gamma_U+\mu+\hat{\rho} & 0 & 0 & 0 & 0 & 0 & 0 & 0 & 0 & 0 & -\tau & 0 & 0 & 0 & 0 & 0 & 0 \\
0 & -\hat{b}_{H1}\delta & \gamma_H+\mu+\hat{\rho} & 0 & 0 & 0 & 0 & 0 & 0 & 0 & 0 & 0 & -\hat{\tau} & 0 & 0 & 0 & 0 & 0 \\
0 & -\hat{b}_{Q1}\delta & 0 & \gamma_H+\mu+\hat{\rho} & 0 & 0 & 0 & 0 & 0 & 0 & 0 & 0 & 0 & -\hat{\tau} & 0 & 0 & 0 & 0 \\
0 & -\hat{b}_{U1}f_{U1}\hat{d}_{U1}\gamma_U & -f_{H1}\hat{d}_{H1}\gamma_H & -f_{Q1}\hat{d}_{Q1}\gamma_Q & \alpha_1 & 0 & 0 & 0 & 0 & 0 & 0 & 0 & 0 & 0 & 0 & -\hat{b}_{U1}f_{U1}\hat{d}_{U1}\gamma_U & -f_{H1}\hat{d}_{H1}\gamma_H & -f_{Q1}\hat{d}_{Q1}\gamma_Q \\
0 & 0 & 0 & 0 & 0 & \sigma+\mu+\rho & 0 & 0 & 0 & 0 & 0 & 0 & 0 & 0 & -\tau & 0 & 0 & 0 \\
0 & 0 & 0 & 0 & 0 & -\sigma & (\hat{b}_{H2}+\hat{b}_{Q2})\delta+\hat{b}_{U2}\gamma_U+\mu+\hat{\rho} & 0 & 0 & 0 & 0 & 0 & 0 & 0 & 0 & -\hat{\tau} & 0 & 0 \\
0 & 0 & 0 & 0 & 0 & 0 & -\hat{b}_{H2}\delta & \gamma_H+\mu+\hat{\rho} & 0 & 0 & 0 & 0 & 0 & 0 & 0 & 0 & -\hat{\tau} & 0 \\
0 & 0 & 0 & 0 & 0 & 0 & -\hat{b}_{Q2}\delta & 0 & \gamma_Q+\mu+\hat{\rho} & 0 & 0 & 0 & 0 & 0 & 0 & 0 & 0 & -\hat{\tau} \\
0 & 0 & 0 & 0 & 0 & 0 & -\hat{b}_{U2}f_{U2}\hat{d}_{U2}\gamma_U & -f_{H2}\hat{d}_{H2}\gamma_H & -f_{Q2}\hat{d}_{Q2}\gamma_Q & \alpha_2 & 0 & -\hat{b}_{U2}f_{U2}\hat{d}_{U2}\gamma_U & -f_{H2}\hat{d}_{H2}\gamma_H & -f_{Q2}\hat{d}_{Q2}\gamma_Q & 0 & 0 & 0 & 0 \\
-\rho & 0 & 0 & 0 & 0 & 0 & 0 & 0 & 0 & 0 & \sigma+\mu+\tau & 0 & 0 & 0 & 0 & 0 & 0 & 0 \\
0 & -\hat{\rho} & 0 & 0 & 0 & 0 & 0 & 0 & 0 & 0 & -\sigma & (\hat{b}_{H2}+\hat{b}_{Q2})\delta+\hat{b}_{U2}\gamma_U+\mu+\hat{\tau} & 0 & 0 & 0 & 0 & 0 & 0 \\
0 & 0 & -\hat{\rho} & 0 & 0 & 0 & 0 & 0 & 0 & 0 & 0 & -\hat{b}_{H2}\delta & \gamma+\mu+\hat{\tau} & 0 & 0 & 0 & 0 & 0 \\
0 & 0 & 0 & -\hat{\rho} & 0 & 0 & 0 & 0 & 0 & 0 & 0 & -\hat{b}_{Q2}\delta & 0 & \gamma+\mu+\hat{\tau} & 0 & 0 & 0 & 0 \\
0 & 0 & 0 & 0 & 0 & \rho & 0 & 0 & 0 & 0 & 0 & 0 & 0 & 0 & \sigma+\mu+\tau & 0 & 0 & 0 \\
0 & 0 & 0 & 0 & 0 & 0 & -\hat{\rho} & 0 & 0 & 0 & 0 & 0 & 0 & 0 & -\sigma & (\hat{b}_{H1}+\hat{b}_{Q1})\delta+\hat{b}_{U1}\gamma_U+\mu+\hat{\tau} & 0 & 0 \\
0 & 0 & 0 & 0 & 0 & 0 & 0 & -\hat{\rho} & 0 & 0 & 0 & 0 & 0 & 0 & 0 & -\hat{b}_{H1}\delta & \gamma_H+\mu+\hat{\tau} & 0 \\
0 & 0 & 0 & 0 & 0 & 0 & 0 & 0 & -\hat{\rho} & 0 & 0 & 0 & 0 & 0 & 0 & -\hat{b}_{Q1}\delta & 0 & \gamma_Q+\mu+\hat{\tau}
\end{bmatrix}$$

R_0 is the spectral radius of FV^{-1}, but because of the complexity of the analytic form of R_0, we exclude it here. Importantly, the fraction of susceptible individuals does not impact the value of R_0 if $0 < S^i < 1$. We use the next generation matrix to compute R_0 numerically in our simulations.

Under simplifying assumptions we can recover the expression for $R_0$for the non-spatial model. For example, excluding demographics ($\mu = 0$) and intervention ($\hat{b}_{H1} = \hat{b}_{H2} = \hat{b}_{Q1} = \hat{b}_{Q2} = 0$), assuming equal migration ($\rho = \tau$) and equal population sizes ($S^{11} + S^{21} = S^{22} + S^{12}$), and assuming all other parameters equal between the two populations (i.e. $\beta_{U1} = \beta_{U2}$, $\beta_{F1} = \beta_{F2}$), the basic reproduction number becomes: $R_0 = \frac{\beta_U}{\gamma_U} + \frac{\beta_F f_U \hat{d}_U}{\alpha}$.

Relaxing some of these assumptions to consider when the populations are not identical but continue to exclude migration ($\tau = \rho = 0$), then the next generation matrix has two non-zero eigenvalues:

$$\frac{\beta_{U1}}{\gamma_U} + \frac{\beta_{F1} f_{U1} \hat{d}_{U1}}{\alpha_1} \qquad \text{and} \qquad \frac{\beta_{U2}}{\gamma_U} + \frac{\beta_{F2} f_{U2} \hat{d}_{U2}}{\alpha_2}$$

the larger of which is the value for R_0.

Indeed, in the absence of migration ($\tau = \rho = 0$) the two populations act independently and all of the analysis of the non-spatial model is recovered for each sub population. In this case, the value for R_0 of the full system is the eigenvalue associated with one population, whichever is largest.

A.5 Control by hospital care: linear relationship between f_H and β_H

As observed in Figure 3 of the main text, there is a critical combination of β_H and f_H such that $R_0 = 1$, independent of β_U and β_F . We therefore investigate the relationship observed analytically and determine the numerical value of these critical values. For simplicity, we find this value in the absence of migration.

In order to simplify R_0 in Figure 3 we assume that all individuals enter the I_H class so no individuals remain in the undetected class and no individuals enter the hospital class ($\hat{b}_H = 1$, $\hat{b}_U = \hat{b}_Q = 0$), thus:

$$R_0 = \frac{\sigma}{(\sigma + \mu)(\delta + \mu)} \left[\beta_U + \frac{\beta_H \delta}{(\gamma_H + \mu)} + \frac{\beta_F}{\alpha} \left(\frac{\delta f_H \hat{d}_H \gamma_H}{\gamma_H + \mu} \right) \right] \quad \text{(A1)}$$

Each line in the figure corresponds to $R_0 = 1$, so we can rewrite Eqn A.5 as:

$$\frac{(\sigma+\mu)(\delta+\mu)}{\sigma} = \beta_U + \frac{\beta_H \delta}{(\gamma_H+\mu)} + \frac{\beta_F}{\alpha}\left(\frac{\delta f_H \hat{d}_H \gamma_H}{\gamma_H+\mu}\right) \tag{A2}$$

Given that all values are fixed on every line except for f_H and β_H, and further that β_F and β_U vary between lines, we define the following constants:

$$\begin{aligned} c_1 &= \frac{\delta}{\gamma_H+\mu} \\ c_2 &= \frac{1}{\alpha}\left(\frac{\delta \hat{d}_H \gamma_H}{\gamma_H+\mu}\right) \\ c_3 &= \frac{(\sigma+\mu)(\delta+\mu)}{\sigma} \end{aligned}$$

Now, we rewrite Eqn. A2 as:

$$c_3 = \beta_U + c_1\beta_H + c_2\beta_F f_H \tag{A3}$$

This expression provides the exact linear relationship between f_H and β_H for fixed values of β_U and β_F.

In addition to this relationship, we also know the exact relationship between β_U and β_F. Specifically, these values were found so that $R_0 = 1.85$ in the absence of hospital care and quarantine ($\hat{b}_U = 1$, $\hat{b}_H = \hat{b}_Q = 0$). Therefore, the relationship between β_U and β_F is given by:

$$1.85 = \frac{\sigma}{(\sigma+\mu)(\gamma_U+\mu)}\left(\beta_U + \frac{\beta_F f_U \hat{d}_U \gamma_U}{\alpha}\right)$$

Defining the following constants:

$$\begin{aligned} c_4 &= 1.85\frac{(\sigma+\mu)(\gamma_U+\mu)}{\sigma} \\ c_5 &= \frac{f_U \hat{d}_U \gamma_U}{\alpha} \end{aligned}$$

we can rewrite the relationship between β_U and β_F as:

$$c_4 = \beta_U + c_5\beta_F \qquad \text{or} \qquad \beta_U = c_4 - c_5\beta_F.$$

Now, we substitute this into the Eqn. A3 relating f_H, β_H, β_F and β_U and we have:

$$c_3 = c_4 - c_5\beta_F + c_1\beta_H + c_2\beta_F f_H \tag{A4}$$

To determine the critical combination of β_H and f_H, we consider "extreme" values of β_F and β_U. First, if we assume that $\beta_F = 0$, then Equation A4 becomes:

$$c_3 = c_4 + c_1\beta_H \qquad \text{or} \qquad \beta_H = \frac{c_3 - c_4}{c_1}.$$

Given the parameters we used, this corresponds to a value of $\beta_H = 0.0542$ which matches the vertical line in Figure 3. On the other extreme, if we assume that $\beta_U = 0$ then:

$$\beta_F = \frac{c_4}{c_5}$$

and Equation A4 becomes:

$$c_3 = c_1\beta_H + \frac{c_2 c_4}{c_5} f_H$$

The intersection of this line and the vertical line is the critical combination of β_H and f_H, which corresponds to ($\beta_H = 0.0542$, $f_H = 0.3501$).
